# Development of an innovative minimally invasive primate spinal cord injury model: A case report

**DOI:** 10.1002/ibra.12117

**Published:** 2023-07-10

**Authors:** Yong‐Min Niu, Jin‐Xiang Liu, Hao‐Yue Qin, Yi‐Fan Liu, Ni‐Jiao Huang, Ji‐Li Jiang, Yan‐Qiu Chen, Si‐Jing Chen, Tao Bai, Chang‐Wei Yang, Yu Cao, Sheng Liu, Hao Yuan

**Affiliations:** ^1^ Institute of Neuroscience Kunming Medical University Kunming Yunnan China; ^2^ Department of Anesthesiology Southwest Medical University Luzhou Sichuan China; ^3^ Yunnan Cancer Hospital The Third Affiliated Hospital of Kunming Medical University Yunnan China; ^4^ Department of Orthopaedic Surgery Affiliated Hospital of Zunyi Medical University Zunyi Guizhou China; ^5^ School of Preclinical Medical Zunyi Medical University Zunyi Guizhou China; ^6^ Nursing School Zunyi Medical University Zunyi Guizhou China; ^7^ School of Preclinical Medical Kunming Medical University Kunming Yunnan China; ^8^ Department of Nuclear Medicine Affiliated Hospital of Zunyi Medical University Zunyi Guizhou China; ^9^ Pharmacology Institute Heidelberg University Heidelberg Germany

**Keywords:** animal models, dorsal 1/4 spinal cord transection, macaque, nonhuman primates, spinal cord injuries

## Abstract

Spinal cord injury (SCI) animal models have been widely created and utilized for repair therapy research, but more suitable experimental animals and accurate modeling methodologies are required to achieve the desired results. In this experiment, we constructed an innovative dorsal 1/4 spinal cord transection macaque model that had fewer severe problems, facilitating postoperative care and recovery. In essence, given that monkeys and humans share similar genetics and physiology, the efficacy of this strategy in a nonhuman primate SCI model basically serves as a good basis for its prospective therapeutic use in human SCI.

## Graphical abstract

We used an innovative 1/4 transection molding method. After the operation, we examined the effect of the molding by magnetic resonance imaging and proposed a new method of primate spinal cord injury molding.

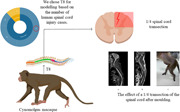

## 1 INTRODUCTION

Spinal cord injury (SCI) is a devastating traumatic condition. Accident rates have increased considerably in recent years due to rapid development of the economy and also technological advancements. About 60% of SCI cases are traumatic, and 80% of patients are younger than 40 years of age.^1^ Many young individuals lose their capacity to work as a result, which places a financial, physical, and mental strain on patients and their families and adversely affects social development. However, there is still a lack of effective treatment for SCI. Therefore, there is an urgent need to identify treatment options that are effective and easy to implement. Currently, the main treatment options are pharmacotherapy (including Chinese, Western, and combined Western and Chinese medicines), rehabilitation, cellular therapy, surgical (including acupuncture, transcranial magnetic, and traditional techniques), and gene therapy, the most novel of which is cellular therapy. It has been the subject of considerable research in recent years.^2^ Cellular therapy includes stem cell therapy, olfactory sheath cell therapy, mesenchymal stem cell therapy, hematopoietic stem cell therapy, and neural stem cell therapy. The treatment used in this case is neural stem cell therapy.^3^ To better investigate neural stem cell therapies, we need more appropriate animal models. Experimental animals are widely used in the study of SCI. Although rodents have the advantages of high genetic similarity to humans, fast reproduction, and ease of breeding, they still differ from humans in terms of the spinal structure.

In contrast, the spinal cord tissues of primates such as marmosets, macaques, and squirrel monkeys are closer to the human spinal cord and more amenable to the study of SCI. Among these, cynomolgus macaques share high similarities with humans in physiology, cognitive abilities, neuroanatomy, and symptoms following injury, and are therefore a good choice of experimental animals for SCI. However, the use of primates has limitations such as higher cost.

In conclusion, the development of more appropriate animal models has clinical value for advancing pertinent research and improving diagnosis and treatment strategies. Therefore, we created a novel primate SCI model utilizing a macaque's spinal cord 1/4 transection, and we verified its efficacy using tests for corridor walking and postoperative hindlimb scoring. This will be highly beneficial for the future of SCI research.

## 2 MATERIALS AND METHODS

### 2.1 Experimental animal selection

Given the highly similar genetics and physiology of macaques to humans, one adult healthy cynomolgus macaque (*Macaca fascicularis*) was selected. This study has been reviewed by the Ethical Review Committee on animal experiments of Kunming Medical University. The ethical declaration number is kmmu20221593 (ethics registration: August 15, 2022).

### 2.2 Options at the SCI level

**Figure 1:**
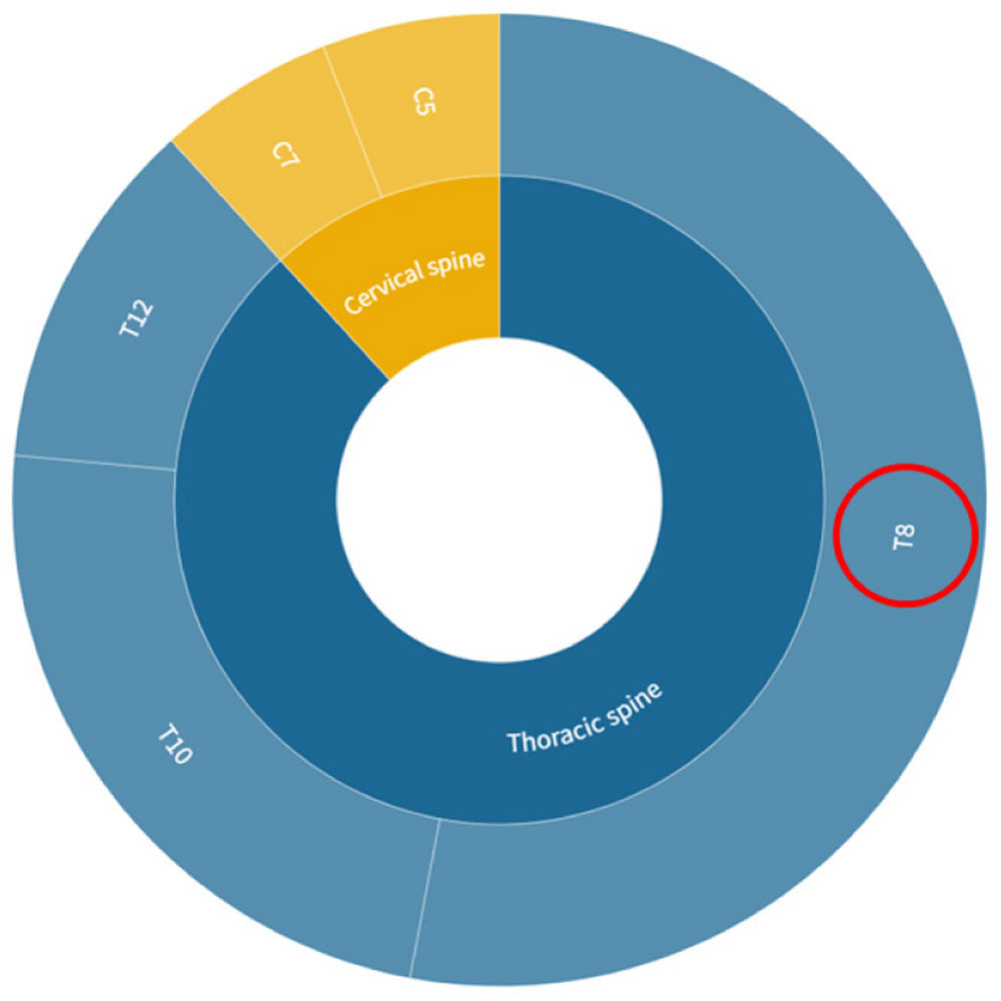
Distribution of the number of people with levels of nerve damage. C, cervical vertebrae; T, thoracic vertebrae. Data were obtained from the PubMed database. [Color figure can be viewed at wileyonlinelibrary.com]

A search of the literature revealed that the commonly used thoracic SCI modeling segments are eighth to twelfth thoracic vertebrae (T8–T12), but no clear studies have been found on the impact of individual segment modeling on experimental outcomes and the characteristics of different segment modeling. Based on the distribution of the number of people with neurological injury levels in epidemiological studies of SCI (Figure 1), it can be seen that a high proportion of patients suffer damage to the T12, T11, T10, and T8 segments of the thoracic spinal cord.^4^ The T8 segment was chosen as the SCI level for this study.

### 2.3 SCI modeling method

According to the experimental objectives, a spinal cord transection injury was chosen as the modeling method for this study.

#### 2.3.1 Marking and positioning

General anesthesia was administered using 1 mL of ketamine hydrochloride (Zhongmao group) and 0.2 mL of chlordiazepoxide xinⅡ (Shengxin, Shengda Animal Drug Co., Ltd.), and after it had taken effect, the patient was placed in the prone position. Following skin preparation, cardiac monitoring, and surgical warming of the bed, the level of T8–T9 was established by feeling the ribs manually and marking the body surface with lines.

#### 2.3.2 Surgical position

The skin and subcutaneous tissues were incised. Then, the fascia was cut and the left vertical spine muscle was left under the periosteum. Next, gauze was used to stop bleeding and the T8–T9, vertebral plate, and articular eminence were revealed.

#### 2.3.3 Creation of a spinal cord transection injury

The dural ligament was peeled away so that the dura mater was exposed. The dura mater was cut open with forceps for about 1 cm to fully expose the spinal cord. The cerebrospinal fluid flowed out and was collected. Small incisions were made at 3‐mm intervals on the dorsal side of the spinal cord with a width of 3 mm and a depth of about 4 mm to create the injury.

#### 2.3.4 Cleaning and dressing

After probing and confirming that the dura mater was not compressed, swollen, or fluctuating, decompression was considered adequate. No active bleeding was detected. After examination, no gauze or instruments were found in the surgical area, and then the muscle layer and the skin layer were gradually sutured to close the incision. Finally, sterile dressing was wrapped and fixed.

#### 2.3.5 Determination of model building

(1) *The 12‐point monkey locomotion scale (MLS)* ^5^: Several assessments have previously been developed to measure locomotion in untrained monkeys after SCI, but forelimb activity has a significant influence on monkey locomotion, making it challenging to quantify hindlimb function in cages. A new 12‐point MLS may be utilized to more accurately evaluate the hindlimb motor function of the monkey while it walks within a 4.5‐m corridor, because the monkey's forelimb suspension and swing are constrained within the corridor.

(2) *Corridor walking test*: The animals were forced to move within a corridor by technical means. Their hip, knee, and ankle joint movements were observed to measure walking ability. The animals' locomotor ability was evaluated by observing how they walk and stand, in addition to observation of their coordinated forward and backward walking, complete body rotation and jumping, and other daily activities, which were scored according to a rating quantification scale.

Line charts were created on the GraphPad to observe the trend changes in the scores to determine the success of model building, based on the data scores of the left and right hind limbs of the macaques.

## 3 RESULTS

**Figure 2:**
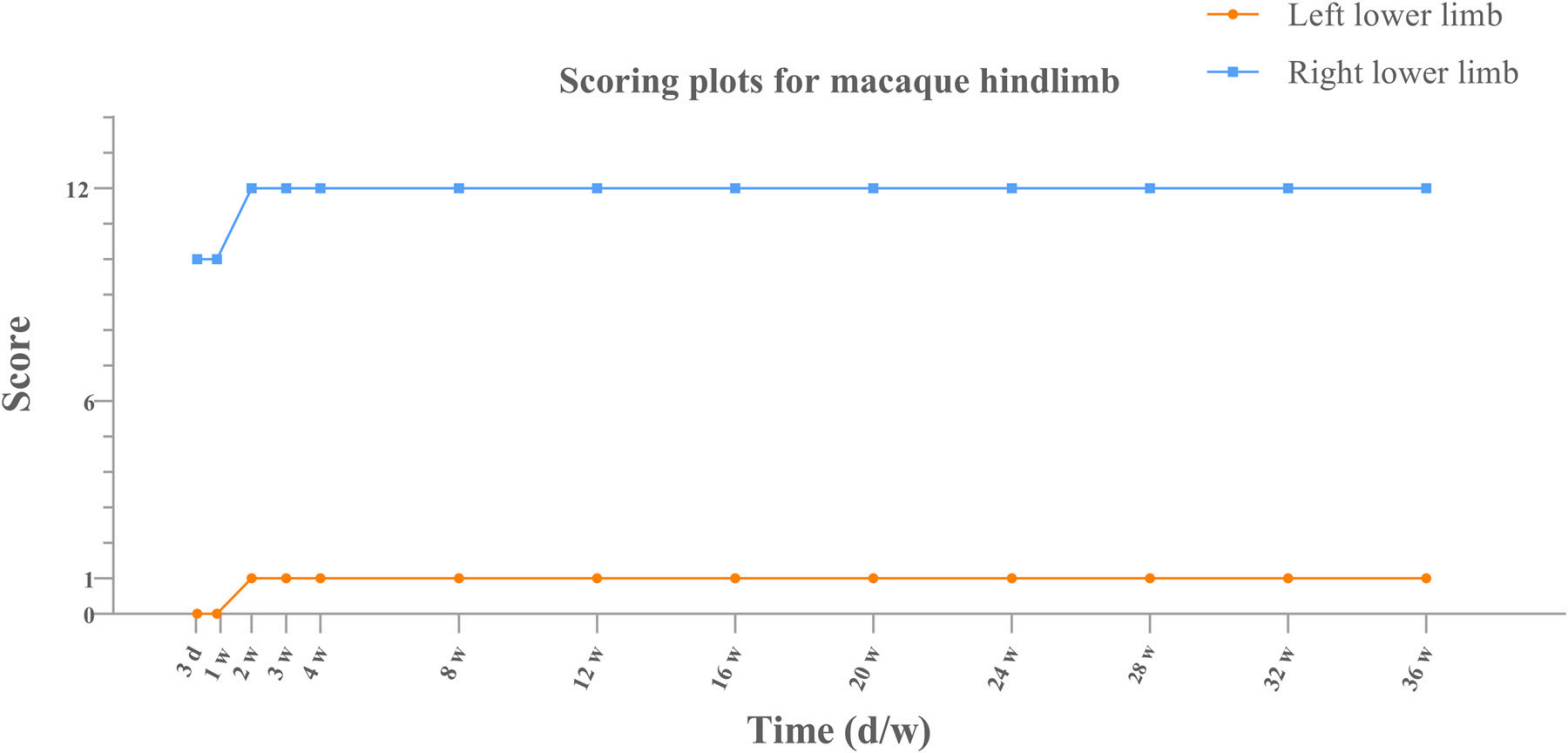
Changes of the monkey hindlimb score after surgical injury. d, day/days; w, week/weeks. [Color figure can be viewed at wileyonlinelibrary.com]

The macaques were studied for a total of 9 months, and the results of the MLS (Table 1) and the corridor walking test are shown in Figure 2. The macaques survived for a total of 9 months, with motor disappearance (symptomatology) of the left limbs consistently present and not improving.

**Table 1:** The 12‐point monkey locomotion scale.

	Score	
	Left	Right
Corridor walking test (walking ability as measured by hip, knee, and ankle movements)		
No voluntary movement in large joints (hip, knee, or ankle)	0	0
Perceptible movement of one to two joints in the hindlimb	1	1
Perceptible movement of three joints	2	2
Vigorous movement of three joints without weight bearing	3	3
Occasional standing without sustainable weight bearing	4	4
Consistent weight support and able to walk with significant deficits	5	5
Standing and walking (3 m in >3 s)	6	6
Standing and walking (3 m in <3 s)	7	5
Coordinated walking forwards and backwards with intact body turning, jumping, and hopping	8	8
Hindlimb digital function		
No bar grasp due to flaccid or spastic digits	0	0
Attempts at digital movement	1	1
Delay in grasping the bar using digits (palm slips from the bar)	2	2
Delay in grasping the bar with a slow release. Digits of the foot are clumsy while standing.	3	3
Effective movement using digits with rapid shift to a relaxed position as balance is attained. Digits open rapidly to perform the next activity.	4	4

*Changes in motor function*: After successful modeling, the left lower limb showed sensory‐motor deficits. It was observed that the left lower limb could not move on its own, and complete contraction of the hip–knee and ankle joints did not occur. The left lower abdomen bulged outwards due to paralysis of the internal oblique and lower transversus abdominis muscle fibers and increased abdominal pressure during breathing.

*Altered sensory function*: Partial loss of right subcostal sensation (mainly pain and warmth) was observed.

**Figure 3:**
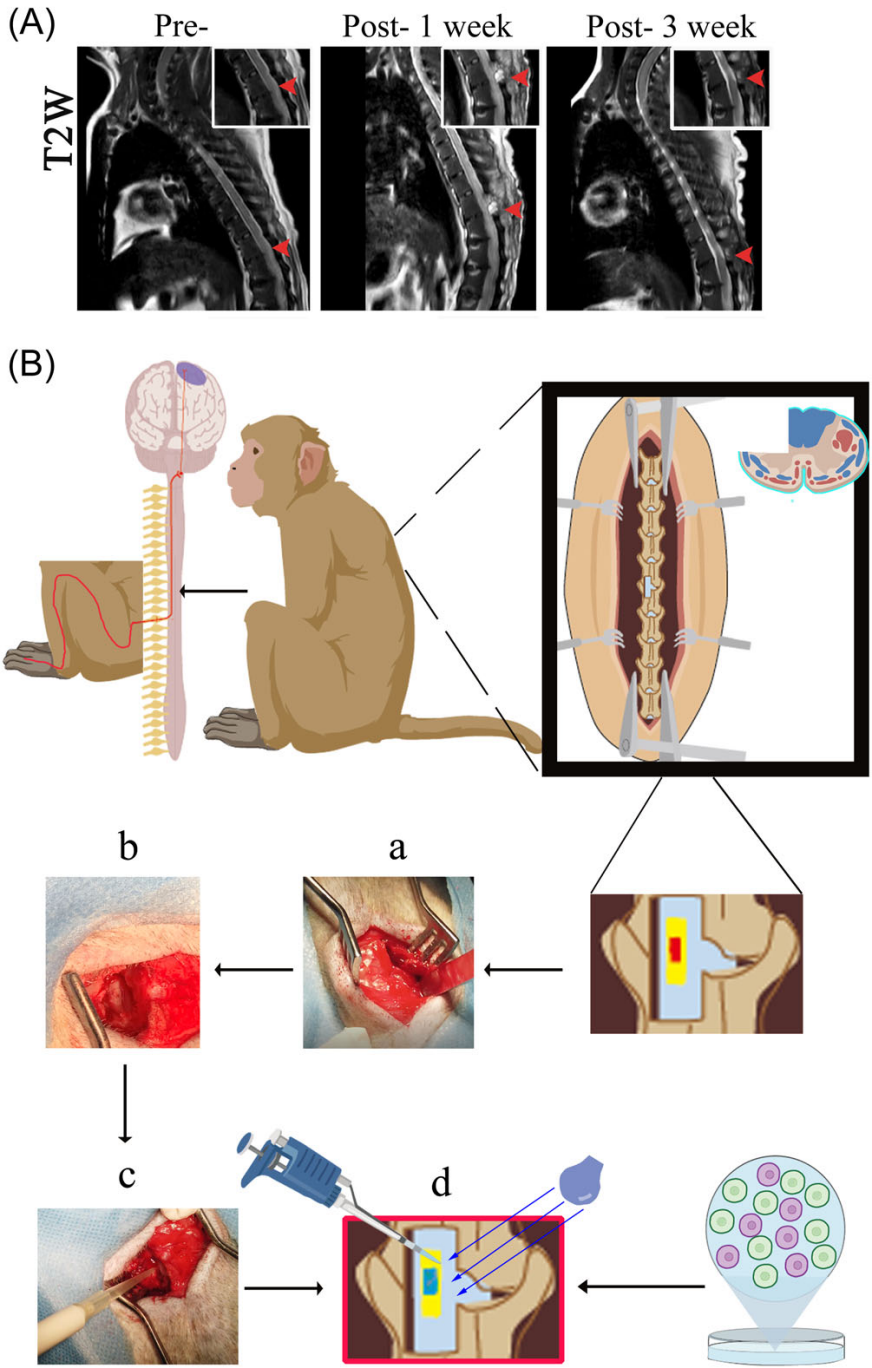
Spinal cord 1/4 transection surgery in the macaque. (A) Magnetic resonance imaging of the macaque spine was divided into “Pre‐” and “Post‐” using surgical manipulation as the boundary. (B) Surgical plan and process of implementation (a: exposing the articular eminence joint, preserving the posterior ligamentous complex; b: a portion of the vertebral plate is removed to create an opening; c: the ligamentum flavum is peeled away to reveal the dura mater; and d: pooled human embryonic neuroepithelial stem cells and human umbilical cord blood stem cells were pipetted into the lesion). [Color figure can be viewed at wileyonlinelibrary.com]

*Altered reflexes*: Partial loss of upper, middle, and lower abdominal wall reflexes on the left side was observed. Magnetic resonance imaging (MRI) results of macaque spine are shown in Figure 3A.

## 4 DISCUSSION

### 4.1 Innovations in animal model selection

Cynomolgus macaques (*M. fascicularis*) were selected as the species for this experiment. The cynomolgus macaque, also known as the long‐tailed macaque, is a species of macaque. The macaque has two major advantages over the use of other experimental animals: they have phylogenetic similarity to nonhuman primates to human subjects and safety and efficacy can be assessed on a small scale before human experimentation.^6^, ^7^ The macaque is currently used in many medical studies such as epidemiology, pharmacology, and toxicology, reproductive physiology, nutrition and metabolism, behavioral and higher neural activity, geriatrics, organ transplantation, and ophthalmology. Macaques have been used in a wide range of neurological studies, including facial perception, prefrontal cortex aging, and so on.^8^, ^9^ The disadvantage is that the experiments are limited by the extremely high costs associated with the care of critically ill animals, which is an issue that will need to be addressed in future studies. It has been reported that the use of contrast‐enhanced ultrasound to quantify spinal cord perfusion in a rhesus monkey model of acute SCI allows for monitoring of SCI, which may be one of the future avenues that can be explored for experimental animal care.^10^

### 4.2 Innovations in SCI level selection

In the mouse model of SCI, most of the experiments were focused on the T10 injured segment.^11^ In the rat SCI model, the T10 injury segment is also used in most experiments.^12^ This is because in rats, successful injury to the T10 segment is marked by tail wagging and retractile fluttering of the hind limbs, both of which can be easily observed, while mice awaken with complete paraplegia. In an experiment aimed at observing the neurological changes caused by SCI, a dog model of SCI was constructed with T10 as the injured segment.^13^ Because SCI in dogs occurs mainly between T10 and L4, T10 is easier to localize. When there is T10 injury in dogs, the symptoms are more similar to those of human SCI.

Due to the difference in anatomy between primates and rodents and canines, the SCI segment selected for this experiment was T8. For example, in the macaque model, the segment selected for the investigation of the repair function of chitosan in SCI was still T8.^14^ Macaques are large and the typical SCI model has a mortality rate of 50%–70% and there is a risk of development of other complications. In contrast, T8 is easy to locate and does not result in a severe loss of limb function or affect the function of the upper abdominal organs, which can effectively reduce mortality. In humans, there is arterial access to T8.^15^ There have been several clinical cases of T8 lesions caused by infectious diseases or cell carcinomas, which have led to SCI.^16^, ^17^ The T8 is the superior part of the spinal cord in the body and is also vulnerable to fracture in the elderly due to osteoporosis.^18^ Many studies in the literature have shown that the T8 segment is more commonly used for the study of SCI. Therefore, selection of T8 as the SCI segment not only ensures the survival of the animals but also allows for discussion and study of the more common sites of injury in humans.

### 4.3 Comparison of macaque SCI and human SCI

In this study, macaques with a 1/4 spinal cord transection injury showed dysfunction in sensation, movement, and partial reflexes. During recovery from SCI treatment, macaques still had a large wound with unhealed trauma 1 week after surgery; the wound of macaques at 3 weeks after operation gradually healed, no obvious damage and abnormality were seen, and the repair morphology was close to that before surgery, accompanied by a full range of motion. After a complete SCI injury in humans, the lesion within the spinal cord progresses, with development of necrosis in the gray matter's nucleus and degeneration of multiple axons in the white matter after 24 h. Additionally, complications could also develop during the recovery process. According to epidemiological research, SCI frequently results in a number of adverse conditions, such as decubitus ulcers, urinary infections, osteoporosis, and so on. Comparative research revealed that decubitus ulcers were more common in patients with traumatic SCI than pneumonia, neuropathic pain, or urinary tract infections. Although the pathophysiology of macaques and humans differs, experimental macaques also experienced postoperative decubitus ulcer symptoms, and both humans and macaques had a relatively high risk of developing complications.

### 4.4 Innovations in mold making and model building

Previously, transverse SCI has been modeled as hemi‐transverse, which can cause respiratory arrest, lung infection, and deep vein thrombosis if not done properly. In this experiment, a dorsal 1/4 spinal cord transection was used as the modeling method (Figure 3B). This allows for the partial transection of the spinal cord to be partially symptomatic and the transplantation of stem cells to be morphologically confirmed for successful transplantation, thus achieving the goal of stem cell therapy for SCI.^19^ At the same time, a model with a 1/4 spinal cord transection can also show a range of symptoms of SCI. An injury at a particular location can simply trigger a loss of function in one lower limb with no effect on the contralateral side.^20^, ^21^ This kind of modeling has advantages in terms of care and long‐term survival. Previous reports in the literature have shown significant destabilization of the spine after total laminectomy, with few reports on the care, survival, and sitting of the subjects.^22^, ^23^, ^24^, ^25^ There is an established need for a more effective and suitable model for behavioral evaluation after treatment and this makes a minimally invasive surgical approach even more necessary. Minimally invasive spinal cord transection is rarely reported in primate studies, so we used a partial laminectomy with a small opening to expose the spinal cord and maximize the preservation of the posterior ligamentous complex of the spine, which is essential for postoperative recovery and postoperative care (Figure 3B).

### 4.5 Problems and solutions

The creation of innovative models requires greater precision, and a thorough grasp of the anatomy of the spinal cord is necessary to successfully develop such a model. MRI plays a crucial role in the diagnostic examination of SCI as it reveals extrinsic compression of the spinal cord and disruption of the disc complex. In addition, it can reveal macroscopic structural evidence of primary intramedullary injury, such as hemorrhage, edema, posttraumatic cystic cavities, and tissue bridges.^26^ Therefore, we performed MRI and three‐dimensional computed tomography reconstructions of the spine of cynomolgus macaques^27^ (Figure 3A).

At the same time, the procedure can reveal many problems, such as the extent of the damage, bleeding, inflammatory spread, postoperative compression of the transplanted cells after coagulation and molding, and so on.^28^, ^29^, ^30^ These can result in secondary SCI, which causes dysfunction and abnormalities below the level of the injury, such as paraplegia and urinary and fecal dysfunction. These complications can lead to difficulties in the evaluation of the SCI model and increase the cost of care, leading to model failure and delayed experimentation.^31^, ^32^ Therefore, the solution is to reduce secondary SCI by conducting more preoperative assessments, preoperative MRI spinal cord assessments, and thorough intraoperative hemostasis, and avoiding spinal cord compression.^33^

## 5 CONCLUSION

In this study, we have described the innovative use of 1/4 transverse SCI molding. This approach reduces severe complications while identifying some of the symptoms of SCI, and reduces the difficulty of postoperative care and recovery. In contrast to the high mortality resulting from complete spinal cord transection, 1/4 spinal cord transection improves the success rate of animal modeling, reducing the incidence of serious complications and the high cost of postoperative care. Furthermore, the unilateral loss of lower limb function demonstrated by 1/4 transection is easier to monitor and compare, making it a better modeling method.

## AUTHOR CONTRIBUTIONS

The experiments were carried out and largely conceptualized by Yong‐Min Niu and Jin‐Xiang Liu. This article was written by Hao‐Yue Qin. Technical support was provided by Ni‐Jiao Huang, Chang‐Wei Yang, Yu Cao, and Yi‐Fan Liu. Animal husbandry was handled by Yan‐Qiu Chen, Si‐Jing Chen, Bai Tao, and Ji‐Li Jiang. Technical guidance was provided by Sheng Liu. Ideas and suggestions were provided by Hao Yuan.

## CONFLICT OF INTEREST STATEMENT

The authors declare no conflict of interest.

## TRANSPARENCY STATEMENT

All the authors affirm this manuscript to be original and based on references.

## ETHICS STATEMENT

This study has been reviewed by the Animal Experimentation Ethics Review Committee of Kunming Medical University. The ethical declaration number is kmmu20221593.

## Data Availability

The data that support the findings of this study are freely available.
